# Waist circumference versus BMI: A cross-sectional study comparing Cardiometabolic Risk association in the Nepalese Population

**DOI:** 10.1371/journal.pone.0353676

**Published:** 2026-07-16

**Authors:** Sushan Gautam, Abhinav Bhattarai, Simran K.C., Roopal Singh, Sanjit Sah, Bishal Budha, Chandra Mani Poudel, Raja Ram Khanal

**Affiliations:** 1 Institute of Medicine, Tribhuvan University, Kathmandu, Nepal; 2 Arthur Riggs Diabetes and Metabolism Research Institute, City of Hope, Duarte, California, United States of America; 3 SR Sanjeevani Hospital, Kalyanpur, Nepal; 4 Manmohan Cardiothoracic Vascular and Transplant Center, Kathmandu, Nepal; University of Diyala College of Medicine, IRAQ

## Abstract

**Background:**

Obesity is a major cardiometabolic risk factor. Although body mass index (BMI) and waist circumference (WC) are established predictors of cardiometabolic risk, their predictive value may vary across ethnic groups. South Asians, including Nepalese individuals, exhibit a distinctive “thin-fat” phenotype characterized by increased central adiposity and higher body fat despite relatively normal BMI levels. Therefore, internationally validated anthropometric cutoffs may not accurately reflect cardiometabolic risk in the Nepalese population. Whether WC better predicts cardiometabolic risk than BMI in Nepalese individuals remains unclear. We conducted a comparative analysis of WC and BMI to evaluate their predictive ability for clinically relevant cardiometabolic outcomes.

**Methods:**

In this cross-sectional study of 678 adults, BMI and waist circumference (WC) were evaluated as anthropometric indicators of cardiometabolic risk in the Nepalese population. Their associations were examined across six clinically relevant outcomes: hypertension, diabetes mellitus, thyroid disease, fatty liver disease (FLD), non-alcoholic fatty liver disease (NAFLD), and dyslipidemia. Comparative performance of BMI and WC was assessed using correlation analysis with biochemical markers, multivariable logistic regression adjusting for key covariates, and receiver operating characteristic (ROC) curve analysis to evaluate their relative predictive and discriminatory ability in this setting.

**Results:**

BMI and waist circumference (WC) were strongly correlated (ρ = 0.75) but demonstrated distinct patterns in cardiometabolic associations. WC showed stronger correlation with triglycerides (ρ = 0.35 vs 0.24 for BMI) and was the only measure significantly associated with hypertension. Both BMI and WC were significantly associated with fatty liver disease (FLD), non-alcoholic fatty liver disease (NAFLD), and dyslipidemia; however, BMI generally showed higher odds ratios per unit increase compared to WC. Predictive performance was comparable between BMI and WC, with similar AUC values in the fair-to-acceptable range (0.6–0.7) across most outcomes. Notably, discrimination improved substantially (>0.8) when BMI and WC were combined with age and sex, yielding good predictive accuracy.

**Conclusion:**

BMI and WC provide complementary information for cardiometabolic risk in the Nepalese population. Combined assessment with age and sex improves risk stratification, indicating that integrating both measures with calibrated thresholds enhances clinical prediction compared with using either alone.

## Introduction

Millions of people worldwide are affected by Non-Communicable Diseases (NCDs) particularly cardiometabolic conditions like Type 2 Diabetes, hypertension and dyslipidemia. Since past few decades, there has been a continuous rise of these conditions in Asian population mainly due to profound changes in food availability, dietary habits and lifestyle [1]. Obesity acts as a primary and synergistic factor in the pathogenesis of cardiovascular diseases (CVD). It directly drives the development of incident cardiometabolic risk factors such as dyslipidemia, type 2 diabetes, hypertension, and sleep disorders [2].

There are a number of anthropometric parameters for assessing obesity like Body Mass Index (BMI), Waist Circumference (WC), Waist to Hip Ratio (WHpR), Waist to Height Ratio (WtHR). The most appropriate measure for assessing CVD risk remains controversial and this ambiguity is further complicated by ethnic differences in the association of fat distribution with CVD risk particularly in South Asian population [3]. BMI has historically served as a primary parameter for measurement of obesity, but the drawback of BMI is that it doesn’t account for fat distribution, particularly visceral adiposity, which plays a chief role in metabolic dysfunction and cardiovascular risk [4]. There is growing evidence that measures of central adiposity like Waist Circumference (WC), Waist to Hip Ratio (WHpR), Waist to Height Ratio (WtHR) are more strongly related to cardiometabolic risk [5,6]. So, relying solely on BMI can lead to under-recognition and misclassification of risk, as many individuals with a normal BMI may still have elevated central adiposity and consequently experience cardiovascular diseases [2]. Despite this heterogeneity, BMI is still most commonly employed parameter for assessing obesity in South Asian countries, including Nepal because of its simplicity and global standardization.

Strikingly, South Asian population is genetically predisposed to accumulate visceral fat than subcutaneous fat which may explain the higher prevalence of cardiometabolic disorders [7]. Although body mass index (BMI) and waist circumference are well-established predictors of cardiometabolic risk, their applicability across ethnic populations remains variable. South Asians, including Nepalese populations, exhibit a distinctive body composition characterized by higher body fat percentage, increased central adiposity, and lower lean muscle mass despite having a relatively normal BMI. This “thin-fat” phenotype predisposes individuals to metabolic disorders such as diabetes, dyslipidemia, hypertension, and fatty liver disease at lower anthropometric thresholds compared to Western populations. Furthermore, Nepalese individuals represent a genetically and geographically diverse population with unique dietary, environmental, and lifestyle influences that may alter the relationship between conventional anthropometric indices and metabolic outcomes. Internationally validated BMI and waist circumference cutoffs, primarily derived from varied populations, may therefore inadequately reflect true cardiometabolic risk in Nepalese adults [8]. Evaluating the comparative association of BMI and waist circumference with cardiometabolic risk factors in the Nepalese population is essential to determine the most reliable predictor and improve ethnicity-specific risk stratification.

Despite the growing global evidence, there is a notable gap in Nepal- specific data. The limited studies conducted in Nepal suggest there is substantial and growing cardiometabolic burden, with over a quarter of country’s population affected by hypertension or pre- hypertension [9]. High blood pressure and high BMI are the driving factors for cardiovascular disease which has emerged as the leading cause of Disability Adjusted Life Years lost in Nepal with National Cardio Vascular Disease (CVD) rates surpassing the South Asian average [10,11]. More recent study like community-based screening in Eastern Nepal has demonstrated a considerable burden of cardiometabolic multimorbidity, with elevated BMI being linked to the occurrence of diabetes and cardiovascular disease [12]. However, the optimal anthropometric tool for identifying these risks in Nepalese population remains understudied. Most studies conducted in Nepal have primarily examined individual risk factors or relied solely on BMI, without directly comparing the predictive utility of WC versus BMI in a head-to-head analysis. This study therefore aimed to compare the associations of BMI and waist circumference with six clinically relevant cardiometabolic outcomes- hypertension, diabetes mellitus, dyslipidemia, fatty liver disease (FLD), non-alcoholic fatty liver disease (NAFLD), and thyroid disease and to evaluate which measures and combinations improves risk discrimination in a sample of Nepalese adults. As secondary objectives, population specific optimal cut-offs for BMI and WC were derived, and the discriminatory performances of additional central adiposity indices (Body Roundness Index [BRI], Waist-to-Height Ratio [WtHR], body fat percentage, and A Body Shape Index [ABSI] were evaluated.

## Methods

### Study design and setting

A cross-sectional observational study was conducted between 13/07/2025–14/11/2025 across outpatient departments of the Institute of Medicine (IOM), Tribhuvan University Teaching Hospital (TUTH), Kathmandu, Nepal, including the Manmohan Cardiothoracic Vascular and Transplant Centre (MCVTC) and the General Practice (GP) Outpatient Department. MCVTC was selected because of its high patient flow with cardiovascular and metabolic comorbidities, including diabetes, hypertension, dyslipidemia, and non-alcoholic fatty liver disease, making it highly relevant for a study focused on cardiometabolic risk prediction. The GP OPD was additionally included as it provides a standardized general health check-up package, ensuring availability of complete anthropometric and biochemical data required for the study objectives.

### Ethical approval

Ethical approval was obtained from the Institutional Review Committee (IRC) of the Institute of Medicine (IOM), Maharajgunj, Kathmandu [Reference number: 717 (6–11) E2 081/082].

### Study population and sampling

Patients visiting the outpatient departments of MCVTC and the General Practice OPD of TUTH who had undergone recent clinical evaluations were included. Anthropometric measurements taken during these visits served as the baseline reference point. Corresponding biochemical and radiological investigations were retrospectively extracted from medical records if they were documented within three-month window of clinical evaluation. A non-probability convenience sampling method was used for participant selection.

Sample size was estimated using ROC analysis methodology, aiming to detect a difference in AUC of 0.10 between BMI and WC, with a power of 80% and 5% significance level. Assuming a prevalence of hypertension of 20% [13,14] and adjusting for up to 4 predictors in logistic regression (BMI, WC, age, sex), the minimum required sample size is estimated to be approximately 400–450 participants. This accounts for adequate statistical power to compare predictive strength and control for confounding. To increase precision and statistical power, we have exceeded the sample size and taken 678 participants.

The formula used is:


$$\begin{array}{c} \text{n}=(({\text{Z}}_{\text{1}}-\alpha /\text{2}\;+\;{\text{Z}}_{\text{1}}-\beta )\;/\;({\text{AUC}}_{\text{1}}\;-\;{\text{AUC}}_{\text{0}}){)}^{\text{2}}\;\times \text{ p}(\text{1}\;-\text{ p}) \end{array}$$

Where:

- Z₁-α/2 = 1.96 (for 95% confidence)

- Z₁-β = 0.84 (for 80% power)

- AUC₁ - AUC₀ = 0.10 (detectable difference in AUCs)

- p = 0.2 (estimated prevalence of outcome)

Inclusion criteria: Only those patients of age ≥ 18 years, whose complete biochemical and anthropometric data were available and who consent to participate were included.

Exclusion criteria: Pregnant women and patients with known history of malignancy or chronic liver disease were excluded.

### Data collection

Written informed consent was taken from all participants. The socio-demographic data and behavioral information (smoking, alcohol intake, diet) were collected through in-person questionnaires.

This study was purely observational and non-interventional. No new diagnoses of hypertension, diabetes mellitus, or thyroid disease were made during the study period. Participants were classified as having these conditions solely based on documented prior diagnoses in their medical records and/or current use of relevant medications. Anthropometric and biochemical measurements obtained during routine clinical evaluation were not used to establish new clinical diagnoses.

The presence of comorbidities was documented based on medical history of diabetes, hypertension and thyroid disease from the patient’s medical records. The biochemical values and radiological findings were taken retrospectively from patient’s medical records which were done within 3 months of the date of the primary clinical encounter where anthropometric data were recorded.

### Anthropometric measurements

Height was measured using a measuring tape and recorded to the nearest 0.1 cm. Participants stood barefoot in an upright position with their back against the wall, heels together, and head maintained in the Frankfort horizontal plane.

Weight was measured using a calibrated spring balance placed on a stable, level surface and recorded to the nearest 1 kg. Participants wore light clothing during measurement.

Body mass index (BMI) was calculated as weight (kg) divided by height squared (m²). Waist circumference (WC) was measured using a non-stretchable measuring tape with participants standing upright in a relaxed posture and feet together. Measurements were taken midway between the lower rib margin and the iliac crest at the end of normal expiration.

In addition to BMI and WC, several derived anthropometric indices were evaluated, including waist-to-height ratio (WtHR), waist-to-BMI ratio (WBMIR), Body Roundness Index (BRI), A Body Shape Index (ABSI), and body fat percentage. These indices were included because they have been proposed as alternative measures of central adiposity and cardiometabolic risk and may provide improved risk discrimination compared with traditional anthropometric measures and may be valuable for South Asian population. BRI estimates body roundness and has been associated with visceral adiposity, whereas ABSI standardizes waist circumference relative to height and BMI to quantify abdominal adiposity independent of overall body size.

BRI and ABSI were calculated according to the following formulas:


$$\begin{array}{c} \text{BRI }=\text{ 364}.\text{2}\;-\text{ 365}.\text{5}\;*\;({\text{1}-[\text{0}.\text{5}*\text{WC}/\pi )}^{\text{2}}\;/\;{\left(\text{0}.\text{5}*\text{height})\text{2}]\right)}^{\text{1}/\text{2}} \end{array}$$

$$\begin{array}{c} \text{ABSI }=\text{ WC}/({\text{BMI}}^{\text{2}/\text{3}}*\;{\text{height}}^{\text{1}/\text{2}}) \end{array}$$

### Biochemical measurements

Biochemical parameters including fasting plasma glucose, lipid profile (total cholesterol, triglycerides, HDL-cholesterol, LDL-cholesterol), liver function test (alanine aminotransferase), renal function tests (urea and creatinine), thyroid function tests (triiodothyronine, tetraiodothyronine, and thyroid-stimulating hormone) and serum uric acid were obtained from hospital laboratory records. All investigations were performed as part of routine clinical evaluation using standard automated analyzers in the central laboratory of Tribhuvan University Teaching Hospital. For study inclusion, laboratory values measured within three months of the clinical visit were considered. As this study involved a retrospective review of existing clinical records, the biochemical results analyzed were those previously reported by the central laboratory in accordance with its established standard operating procedures. Fasting venous blood samples (minimum 8–12 h fast) were collected in the morning in specialized vacutainers and processed within three hours of collection. All assays were performed in automated analyzers (Cobas series, Roche Diagnostics) in the ISO-certified central laboratory of TUTH, which participates in an external quality assurance program including External Quality Assessment Services (EQAS) such as Christian Medical College Vellore, India. Fasting plasma glucose was analyzed using the enzymatic hexokinase method. Lipid parameters, including total cholesterol, triglycerides, HDL-cholesterol, and LDL-cholesterol, were assessed using enzymatic colorimetric assays. Liver enzyme alanine aminotransferase (ALT) was measured by the IFCC-recommended kinetic UV method, while thyroid function tests (T3, T4, and TSH) were evaluated using electrochemiluminescence immunoassay (ECLIA). Serum uric acid was determined using the uricase-PAP method.

### Radiological assessment

Fatty liver disease was assessed using abdominal ultrasonography as part of routine clinical evaluation. Ultrasonographic findings documented in patients’ medical records were reviewed, and fatty liver was diagnosed based on standard sonographic criteria. Where available, fatty liver was graded as mild (grade 1), moderate (grade 2), or severe (grade 3) according to the radiologist’s report. Consistent with the approach applied to biochemical data, only ultrasonography reports documented within three months of the clinical visit (i.e., the date of anthropometric measurement) were included in hepatic steatosis classification. Sonographic diagnosis of fatty liver was based on standard B-mode ultra-sonographic criteria: increased hepatic echogenicity relative to the renal cortex and posterior attenuation of acoustic beam [15] as interpreted by a qualified radiologist.

### Cardiometabolic risk outcomes

The cardiometabolic risk outcomes evaluated in this study included hypertension, diabetes mellitus, dyslipidemia, fatty liver disease (FLD), non-alcoholic fatty liver disease (NAFLD), and thyroid disease.

A condition-specific classification approach was adopted for cardiometabolic outcomes. Dyslipidemia was operationally defined as a binary variable derived from NCEP ATP III lipid thresholds, where the presence of any abnormal lipid parameter (LDL-C ≥ 100 mg/dL, Total Cholesterol ≥ 200 mg/dL, Triglycerides ≥ 150 mg/dL, or HDL-C < 40 mg/dL or males and <50 mg/dL for females) was classified as dyslipidemia [16]. This threshold is widely used in epidemiological and clinical studies for its explicit laboratory cut-offs suitable for population-based classification rather than guidelines designed for treating hyperlipidemia. Diabetes mellitus, hypertension, and thyroid disease were defined based on documented prior physician diagnosis or current pharmacological treatment. This was done because diabetes mellitus, hypertension, and thyroid disease are clinical diagnoses that cannot be established from a single laboratory value alone. The diagnostic criteria for each as per the American Diabetes Association, the American Heart Association, and the American Thyroid Association respectively require either repeat biochemical confirmation, oral glucose tolerance testing, HbA1c correlation [17], multiple blood pressure measurements across separate visits [18,19], or clinical symptom correlation before a diagnosis can be assigned [20,21]. Applying a single retrieved fasting glucose, blood pressure reading, or TSH value from a retrospective record to classify participants as diseased or non-diseased would therefore not satisfy the diagnostic requirements for these conditions and would introduce significant misclassification.

Fatty liver disease was identified based on ultrasonographic evidence documented in radiology reports. NAFLD was defined as ultrasonographically detected fatty liver in the absence of significant alcohol consumption or secondary causes, based on available clinical documentation. Ultrasound findings of within-three months was used for the diagnosis.

### Statistical analysis

Statistical analyses were performed using RStudio (version 2024.12.0 + 467). Descriptive statistics were generated for all continuous and categorical variables. Normality was assessed using the Shapiro–Wilk test. As continuous variables were not normally distributed, data were summarized as median and interquartile range (IQR; Q1–Q3), while categorical variables were reported as frequencies and percentages. Statistical significance was defined as a two-sided p-value < 0.05.

Associations between anthropometric measures, including body mass index (BMI) and waist circumference (WC), and blood parameters were evaluated using Spearman’s rank correlation coefficients. Simple linear regression analyses were subsequently performed to further characterize significant associations and assess the direction and magnitude of linear relationships.

Participants were stratified according to cardiometabolic risk groups, including hypertension (HTN), diabetes mellitus (DM), thyroid disease, fatty liver disease (FLD), non-alcoholic fatty liver disease (NAFLD), and dyslipidemia. Differences in BMI, WC, and blood parameters between two-group comparisons were assessed using the Wilcoxon rank-sum test, whereas comparisons involving more than two groups were evaluated using the Kruskal–Wallis test.

Binary logistic regression analyses were conducted to evaluate the associations of BMI and WC with cardiometabolic outcomes. Results were reported as odds ratios (ORs) with 95% confidence intervals (CIs). Multivariable logistic regression models were adjusted for age, sex, smoking status, alcohol consumption, and dietary factors to account for potential confounding.

The predictive performance of BMI and WC for cardiometabolic outcomes was assessed using receiver operating characteristic (ROC) curve analysis and quantified by the area under the curve (AUC). ROC analyses were performed for anthropometric measures alone and for models incorporating significant covariates. Optimal cut-off values were determined using Youden’s J statistic.

To determine whether alternative obesity-related indices provided superior predictive performance WtHR, WBMIR, BRI, ABSI, and body fat percentage were evaluated using the same logistic regression and ROC analysis framework. The predictive abilities of these indices were compared with those of BMI and WC across all cardiometabolic outcomes.

## Results

### Patient demographics and descriptives

There were altogether 678 patients comprised on 292 males and 386 females. All continuous variables were non-normal in distribution. The median age was 45 (Q1: 33, Q3: 55). Out of them 97 were smokers, 131 consumed alcohol, and 646 followed a non-vegetarian diet. The prevalence of hypertension, diabetes mellitus, and thyroid disease were 15.7%, 6.2%, and 8.7% respectively. Table 1 summarizes the demographics and descriptive statistics. No significant difference in the waist circumference between males and females (p = 0.136)

**Table 1:** Patient demographics and descriptives statistics of BMI, Waist, Age, and blood parameters.

Descriptive summary of categorical variables			Descriptive statistics of continuous variables		
Category	N	%	Variable	Median (Q1-Q3)	
Sex			Age	45 (33 - 55)	
Female	386	56.9	Male	43 (30–55)	P = 0.367
			Female	45 (35 - 55)	
Male	292	43.1	BMI	25.5 (22.9 - 28.7)	
Alcohol					
No	547	80.7	Waist circumference (WC)	90 (82 - 95)	
Yes	131	19.3	Male	90 (84 - 96)	P = 0.148
			Female	90 (80–95)	
Diabetes mellitus			Glucose	4.9 (4.5 - 5.3)	
No	636	93.8	Triglyceride	1.3 (0.8 - 1.9)	
Yes	42	6.2	HDL	1.1 (1 - 1.3)	
Diet			LDL	3.1 (2.5 - 3.7)	
Vegetarian	32	4.7	Cholesterol	4.7 (4 - 5.38)	
Non-vegetarian	646	95.3	ALT	26 (19 - 39)	
Hypertension			UA	304 (256 - 364)	
No	570	84.3	T3	4.52 (4.07 - 4.94)	
Yes	106	15.7	T4	12.34 (11.29 - 13.44)	
Smoking			TSH	1.92 (1.29 - 3.06)	
No	384	85.7	Urea	3.5 (2.9 - 4.3)	
Yes	292	14.3	Creatinine	60 (53 - 70)	
Thyroid disease			Vitamin B12	339 (196 - 448)	
No	618	91.3	Vitamin D	20.7 (13.35 - 24.2)	
Yes	59	8.7			

Age: years, waist: cm, glucose, triglycerides, HDL, LDL, cholesterol, urea, creatinine: mmol/L, ALT: U/L, uric acid (UA): µmol/L, triiodothyronine (T3): pmol/L, tetraiodothyronine (T4): µg/dL, thyroid stimulating hormone (TSH): mIU/L, vitamin B12: pg/mL, vit D: ng/mL.

### Correlation of BMI and waist circumference (WC) with blood parameters

**Fig 1:**
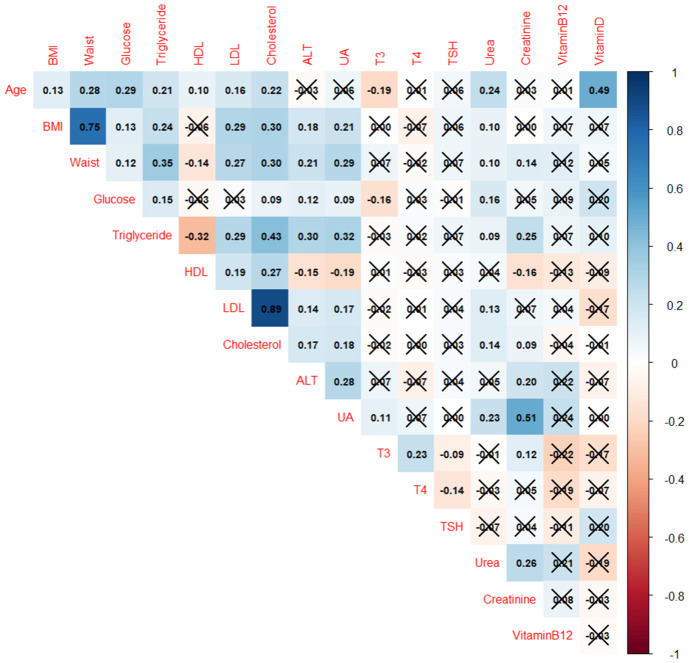
Correlation coefficient heatmap. Non-significant correlations are crossed.

BMI and WC were highly correlated (rho = 0.75). WC had a higher correlation with age than BMI (rho = 0.28 vs. 0.13). With glucose, there was a weak positive correlation of both BMI (rho = 0.13) and WC (rho = 0.12). Both BMI and WC correlated significantly with lipid parameters: triglyceride, LDL, and total cholesterol, but not with HDL. WC had a higher correlation with triglycerides than BMI (rho 0.35 vs. 0.24). Likewise, both BMI and WC correlated significantly with ALT, UA, and urea with similar correlation coefficients. Overall, the highest correlation with any blood parameter observed was between WC and triglyceride. Fig 1 displays the correlation matrix of all variables.

### Predictability of BMI and WC to levels of blood parameters

The simple linear regression showed no linear relationship and predictability of BMI and WC to glucose levels. WC showed predictive ability to all lipid parameters: triglyceride, LDL, HDL, and cholesterol, whereas BMI was only related with HDL and LDL. Rise in liver enzyme ALT was related only to WC and not BMI. Both BMI and WC could predict uric acid levels. Additionally, renal parameters urea and creatinine was only predictable by WC and not BMI. Table 2 summarizes the linear relationship of all parameters with BMI and WC. For parameters which could be predicted by both BMI and WC, the change in parameters were much higher followed by elevation in 1 unit WC as compared to BMI.

**Table 2:** Simple linear regression analysis of BMI and WC with blood parameters.

Independent	Dependent	Slope	p-value	Dependent	Independent	Slope	p-value
Age	BMI	0.025	0.37	Age	WC	0.201	<0.001
BMI	WC	2.024	<0.001	WC	BMI	0.28	0.56
	Glucose	0.003	0.62		Glucose	0.01	0.01
	Triglycerides	0.009	0.08		Triglycerides	0.02	0.03
	HDL	−0.002	0.02		HDL	−0.004	0.02
	LDL	0.005	0.11		LDL	0.02	0.08
	Cholesterol	0.007	0.06		Cholesterol	0.03	0.09
	ALT	0.094	0.28		ALT	0.30	0.02
	UA	0.781	0.01		UA	2.29	0.08
	T3	−0.001	0.66		T3	0.003	−0.001
	T4	−0.008	0.16		T4	−0.001	−0.002
	TSH	−0.107	0.74		TSH	0.01	0.003
	Urea	0.002	0.72		Urea	0.02	0.010
	Creatinine	−0.012	0.89		Creatinine	0.27	0.02
	Vitamin B12	14.812	0.16		Vitamin B12	4.94	0.001
	Vitamin D	0.831	0.17		Vitamin D	0.27	0.02

Slope is equivalent to the change in dependent variable with 1 unit change in independent variable. For example: 1 cm increase in waist circumference will result in increase in uric acid by 2.3 µmol/L and 10 cm by 23 µmol/L.

### Differences of BMI and WC between various cardiometabolic groups

BMI and WC were significantly higher in patients with HTN (p < 0.001) but did not differ based on DM status (p = 0.350 and 0.159). BMI was higher in patients with thyroid disease (p = 0.03), however there was no difference in WC based on thyroid status (p = 0.656). Overall, both BMI and WC were higher in FLD patients than those without FLD. The values significantly differed between FLD grades. While higher FLD grades were observed to have higher WC, they were not statistically significant. Similar variation was observed with BMI with insignificant statistics. Overall, both BMI and WC were associated with all cardiometabolic risk groups except diabetes mellitus (Table 3).

**Table 3:** Differences of BMI and WC between different cardiometabolic risk groups.

Group	Status	BMI (Kg/m^2^)	p-value	Waist (cm)	p-value
HTN	Yes (N = 106)	26.81 (25.00 - 29.55)	<0.001^a^	93.00 (89.00–97.00)	<0.001^a^
	No (N = 570)	25.30 (22.51–28.36)		89.00 (81.00–95.00)	
Diabetes	Yes (N = 42)	26.19 (24.09–28.34)	0.350^a^	92.00 (88.00–95.50)	0.159^a^
	No (N = 636)	25.46 (22.83–28.73)		90.00 (81.50–95.00)	
Thyroid disease	Yes (N = 59)	27.19 (24.52–29.55)	0.037^a^	89.50 (82.00–94.75)	0.656^a^
	No (N = 618)	25.42 (22.87–28.38)		90.00 (82.75–95.25)	
Fatty liver disease	Grade 3 (N = 8)	30.00 (28.20–31.20)	<0.001^b^	104.00 (96.00–106.00)	<0.001^b^
	Grade 2 (N = 53)	29.60 (26.10–32.50)		101.00 (90.00–107.00)	
	Grade 1 (N = 226)	27.40 (25.20–30.10)		95 (90.00–99.00)	
	No (N = 391)	24.00 (21.90–26.30)		86 (78.00–92.00)	
NAFLD	Yes (N = 227)	28.06 (25.57–30.65)	<0.001^a^	95.00 (90.00–100.25)	<0.001^a^
	No (N = 451)	24.51 (22.04–26.78)		88.00 (80.00–93.00)	
Dyslipidemia	Yes (N = 536)	25.81 (23.45–28.88)	<0.001^a^	90.00 (84.00–96.00)	<0.001^a^
	No (N = 142)	22.52 (20.20–24.17)		80.00 (75.00–86.00)	

Values represented in Median (Q1 – Q3), a: Wilcoxon Rank-Sum test, b: Kruskal-Wallis test; post-hoc test in supplementary.

### Odds of HTN, DM, Thyroid disease, FLD, NAFLD, and dyslipidemia based on BMI and WC

**Fig 2:**
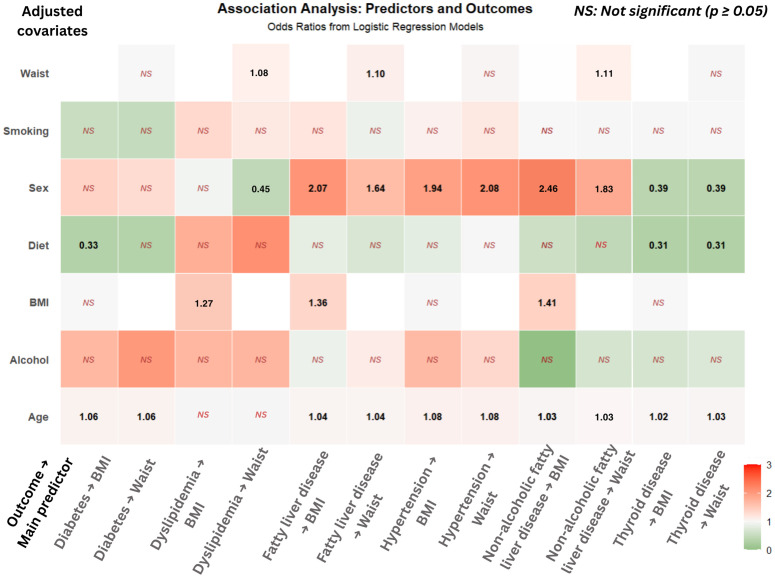
Heatmap diagram of covariate-adjusted ORs of multivariate regression analysis.

In the univariate analysis, there was no significant odds of DM and thyroid disease for both BMI and WC. For HTN, only WC had significant odds (OR: 1.04) however, this was modest. Significant odds for FLD, NAFLD, and dyslipidemia was observed for both BMI and WC, however, the OR was higher for BMI based analysis than WC. Similar results were seen when BMI and WC were adjusted for age, sex, smoking status, alcohol use, and diet(vegetarian vs. non-vegetarian). The results have been summarized in table 4. Among the covariates, age was significantly associated with all outcomes with small ORs (1.03–1.08). Sex (male) was significantly associated with HTN, FLD and NAFLD (ORs: 2.07^BMI^ 1.94^Waist^, 1.94^BMI^ 2.08^Waist^, and 2.86 respectively). In contrast male sex was associated with a lower odds for thyroid disease (0.39^both models^). Interestingly, smoking and alcohol was not associated with any outcomes. Non-vegetarian diet was associated with lower odds of diabetes (OR=0.33^BMI^) and thyroid disease (OR=0.31 ^both models^). Adjusted ORs are displayed in Fig 2. Overall, this indicated that BMI is associated with higher odds of FLD, NAFLD, and dyslipidemia than WC. Male patients are more likely to develop HTN, FLD, and NAFLD while less likely to develop thyroid disease.

**Table 4:** Univariate and multivariate logistic regression analysis of BMI and WC with risk groups.

	Univariate Logistic Regression (Unadjusted)				Multivariate Logistic Regression (Adjusted)^a^		
Predictor	Outcome	Odds Ratio	95% CI	p-value	Odds Ratio	95% CI	p-value
BMI	Hypertension	1.01	0.99-1.03	0.269	1.01	1-1.04	0.091
	Diabetes	1.00	0.94-1.02	0.990	1.00	0.94-1.02	0.886
	Thyroid disease	1.00	0.97-1.02	0.751	1.00	0.96-1.02	0.944
	Fatty liver	**1.33**	**1.26-1.4**	**<0.00001**	**1.36**	**1.29-1.44**	**<0.00001**
	NAFLD	**1.30**	**1.23-1.37**	**<0.00001**	**1.41**	**1.33-1.51**	**<0.00001**
	Dyslipidemia	**1.25**	**1.18-1.33**	**<0.00001**	**1.27**	**1.17-1.38**	**<0.00001**
Waist circumference	Hypertension	**1.04**	**1.02-1.07**	**<0.00001**	1.03	1-1.05	0.070
	Diabetes	1.02	0.99-1.06	0.260	1.00	0.97-1.04	0.882
	Thyroid disease	0.99	0.96-1.02	0.642	0.99	0.96-1.02	0.453
	Fatty liver	**1.11**	**1.08-1.14**	**<0.00001**	**1.10**	**1.08-1.13**	**<0.00001**
	NAFLD	**1.08**	**1.06-1.11**	**<0.00001**	**1.11**	**1.08-1.15**	**<0.00001**
	Dyslipidemia	**1.07**	**1.05-1.1**	**<0.00001**	**1.08**	**1.04-1.12**	**<0.00001**

a: Covariates adjusted were age, sex, alcohol, diet, and smoking.

### Performance of BMI versus WC in classifying cardiometabolic risk outcomes

**Fig 3:**
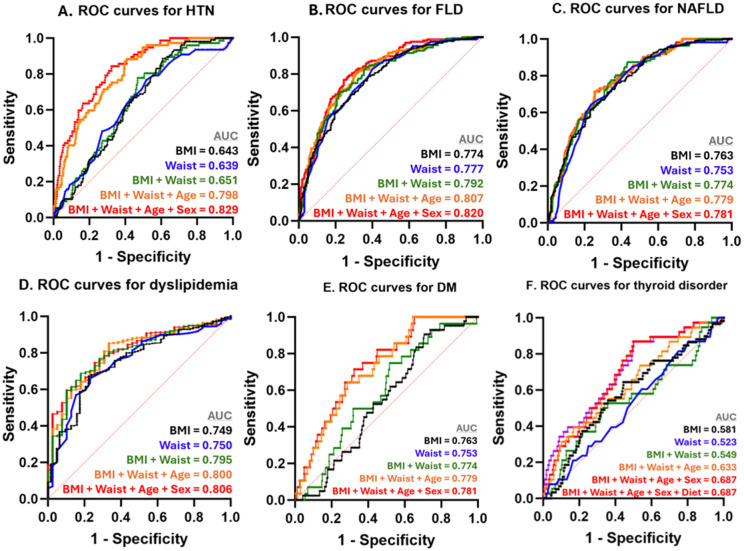
ROC curves of analysis of BMI and WC models to evaluate the accuracy with outcomes.

Hypertension: BMI and WC alone had similar AUCs (0.643 and 0.639; fair) for hypertension. When combined, the increment was mild (0.651). In contrast, there was a sharp increment when age and sex together with BMI and WC (AUC = 0.829; good) (Fig 3A).

Diabetes: Both BMI and WC had a poor AUC (0.543 and 0.579) to classify diabetes. The highest AUC was 0.736 when BMI and WC were combined and supplemented with age and diet (Fig 3E).

Thyroid: Accuracy of both BMI and WC in classifying thyroid disease was low (0.581 and 0.523). Increase in AUC was observed when BMI and WC together were combined with age, sex, and diet (0.687;Fair).

NAFLD: BMI and WC alone had similar AUCs (0.763 and 0.753; acceptable) for NAFLD. When combined, the increment was mild (0.774). The highest AUC was 0.781 when combined together with age and sex (Fig 3C).

FLD: BMI and WC alone had similar AUCs (0.774 and 0777; acceptable) for FLD. When combined, the increment was mild (0.792). In contrast, there was a yield of AUC 0.820 when age and sex were combined with BMI and WC for FLD, indicating good accuracy (Fig 3B).

Dyslipidemia: BMI and WC alone had similar AUCs (0.749 and 0.750; acceptable) for dyslipidemia. When combined, the increment was mild (0.795). The highest AUC was 0.806 when combined together with age and sex (Fig 3D).

Overall, BMI and WC standalone had good accuracy only for FLD, NAFLD, and dyslipidemia and accuracy improved when combining associated covariates (Fig 3).

### Optimal cut-offs of BMI and WC for risk outcomes

Optimal BMI and waist circumference (WC) cut-offs for cardiometabolic risk were derived from ROC analysis using Youden’s J statistic (S1 Table). For hypertension and diabetes, BMI thresholds of 24.1–24.4 kg/m² showed high sensitivity (88.7%–90.5%) but low specificity (<40%). Thyroid disease required a higher BMI (25.95 kg/m²) with moderate sensitivity and specificity (64.4% and 55.4%). Fatty liver disease and NAFLD showed the strongest discrimination, with BMI 25.1–26.0 kg/m² and WC 88.5–93.5 cm. Dyslipidemia was best predicted by BMI 24.8 kg/m². Overall, BMI ~ 25 kg/m² and WC ~ 88–93 cm indicate substantially increased cardiometabolic risk, especially for fatty liver disease.

### Association of additional obesity metrics with cardiometabolic risk outcomes

Table 5 shows that alternative obesity indices displayed varying predictive power for cardiometabolic risks. BRI was the strongest predictors, showing significant associations and higher AUCs for fatty liver (0.746) and NAFLD (0.739), outperforming BMI and WC. WtHR was linked to dyslipidemia (OR = 2.82, AUC = 0.702). It showed a higher odds of FLD and NAFLD than BMI or WC, however, the AUCs were lower. WBMIR showed inverse associations with fatty liver and NAFLD, while ABSI had weak and non-significant results (AUC ~ 0.5). Overall, BRI demonstrated superior discriminatory accuracy for HTN, FLD, and NAFLD while WtHR stood superior for its association with dyslipidemia. These findings indicated that these obesity metrics might have varying implications for different cardiometabolic risks.

**Table 5:** Logistic regression and ROC analysis of additional obesity metrics with risk outcomes.

	Univariate Logistic Regression (Unadjusted)				Multivariate Logistic Regression (Adjusted)^*^		
Predictor	Outcome	Odds Ratio	95% CI	p-value	Odds Ratio	95% CI	p-value
Waist circumference to height ratio (WtHR)	Hypertension	**1.80**	**1.28-2.57**	**0.0008**	**1.68**	**1.10-2.62**	**0.020**
	Diabetes	1.48	0.87-2.48	0.140	1.18	0.66-2.12	0.587
	Thyroid disease	1.13	0.71-1.77	0.605	0.78	0.47-1.27	0.320
	Fatty liver	**3.63**	**2.61-5.18**	**<0.0001**	**4.45**	**2.99-6.85**	**<0.0001**
	NAFLD	**3.08**	**2.20-4.41**	**<0.0001**	**4.44**	**2.99-6.85**	**<0.0001**
	Dyslipidemia	**3.43**	**2.08-5.82**	**<0.0001**	**3.03**	**1.74-5.36**	**<0.0001**
Waist circumference to BMI ratio (WBMIR)	Hypertension	**0.49**	**0.24-0.96**	**0.040**	**0.16**	**0.06-0.38**	**<0.0001**
	Diabetes	1.07	0.37-2.96	0.899	0.78	0.24-2.47	0.675
	Thyroid disease	0.70	0.28-1.73	0.445	0.94	0.35-2.48	0.895
	Fatty liver	**0.44**	**0.25-0.75**	**0.002**	**0.27**	**0.14-0.51**	**<0.0001**
	NAFLD	**0.27**	**0.14-0.50**	**<0.0001**	**0.19**	**0.08-0.40**	**<0.0001**
	Dyslipidemia	**0.26**	**0.10-0.62**	**0.002**	**0.30**	**0.12-0.75**	**0.010**
Body Roundness Index (BRI)	Hypertension	**1.30**	**1.11-1.52**	**0.001**	**1.26**	**1.04-1.54**	**0.021**
	Diabetes	1.18	0.93-1.48	0.162	1.06	0.81-1.37	0.655
	Thyroid disease	1.05	0.85-1.29	0.635	0.89	0.70-1.11	0.304
	Fatty liver	**1.77**	**1.52-2.08**	**<0.0001**	**1.93**	**1.62-2.35**	**<0.0001**
	NAFLD	**1.62**	**1.39-1.89**	**<0.0001**	**1.91**	**1.62-2.35**	**<0.0001**
	Dyslipidemia	**1.90**	**1.46-2.52**	**<0.0001**	**1.79**	**1.34-2.42**	**<0.0001**
A Body Shape Index (ABSI)	Hypertension	1.03	0.70-1.54	0.868	0.59	**0.36-0.94**	**0.028**
	Diabetes	1.42	0.77-2.67	0.266	1.02	0.54-1.99	0.954
	Thyroid disease	0.96	0.57-1.63	0.870	0.82	0.48-1.41	0.805
	Fatty liver	1.30	0.95-1.79	0.103	1.04	0.74-1.46	0.805
	NAFLD	1.00	0.71-1.40	0.982	0.91	0.62-1.33	0.618
	Dyslipidemia	1.02	0.60-1.70	0.947	0.86	0.49-1.50	0.601
Body fat%	Hypertension	1.01	1.00-1.03	0.127	1.01	1.00-1.03	0.091
	Diabetes	1.01	0.98-1.02	0.465	1.00	0.95-1.02	0.886
	Thyroid disease	1.01	1.00-1.03	0.110	1.00	0.97-1.02	0.945
	Fatty liver	**1.08**	**1.06-1.11**	**<0.0001**	**1.29**	**1.23-1.36**	**<0.0001**
	NAFLD	**1.09**	**1.07-1.12**	**<0.0001**	**1.33**	**1.26-1.41**	**<0.0001**
	Dyslipidemia	**1.08**	**1.05-1.12**	**<0.0001**	**1.22**	**1.14-1.31**	**<0.0001**

*: Covariates adjusted for age, sex, alcohol, diet, and smoking.

## Discussion

In this hospital-based cohort of 678 adults, BMI and waist circumference (WC) were strongly associated with hypertension, dyslipidemia, and fatty liver disease (FLD/NAFLD), but less predictive of diabetes. BMI and WC were highly correlated (rho = 0.75) yet conveyed distinct signals: WC correlated more with age (rho 0.28 vs 0.13) and triglycerides (rho 0.35 vs 0.24). Both independently predicted FLD/NAFLD in multivariable models, while WC’s association with hypertension was modest and attenuated after adjustment. These results highlight a complementary role for BMI and WC, with WC showing greater metabolic specificity.

### Central adiposity indices (WC, WtHR, BRI) vs. BMI – quantitative comparisons

Our findings support that central adiposity measures outperform BMI for metabolic outcomes, with WtHR and WC better predicting diabetes, CVD, and overall risk; a WtHR of 0.5 is proposed as a simple global threshold [5,22]. In a Colombian cohort (n = 29,236), Montoya Castillo et al. found WtHR was the strongest predictor of cardiometabolic risk (OR 3.04 unadjusted; OR 1.99 adjusted for WC, OR 2.48 for BMI), while BMI risk plateaued near 30 kg/m² and WtHR showed a linear relationship [23]. These patterns mirror our findings, with central adiposity indices showing more linear, sensitive associations with risk, while BMI plateaus at higher values; accordingly, WC and derived measures (BRI, WtHR) often provide incremental discrimination beyond BMI alone.

At the discrimination level, our AUCs align with prior reports. We observed acceptable-to-good AUCs for BMI and WC in FLD and NAFLD (FLD: BMI 0.774, WC 0.777; NAFLD: BMI 0.763, WC 0.753), with combined models plus age/sex improving performance into the good range (FLD AUC 0.820). Similarly, Suwała et al. reported comparable sex-specific differences (men: BMI 0.816 vs WC 0.804; women: WC 0.762 vs BMI 0.739) and proposed intermediate BMI and WC cut-offs to optimize cardiovascular risk discrimination [24]. Our optimal cut-offs (BMI ≈ 25–26 kg/m²; WC ≈ 88–93.5 cm) align with cross-population risk thresholds for mid-20s BMI and high-80s/low-90s WC.

### Hypertension – joint contribution of BMI and WC and comparison with other cohorts

Hypertension was the most common outcome (15.7%), with higher BMI and WC in affected participants (BMI 26.8 vs 25.3 kg/m²; WC 93 vs 89 cm; p < 0.001), though only WC showed a significant unadjusted association (OR 1.04 per cm). Adjusting for age and sex reduced both effects but improved discrimination (AUC ~ 0.64 → 0.829). This pattern, WC adding value while anthropometry requires demographic adjustment, aligns with studies highlighting abdominal obesity as a key driver of elevated blood pressure and mirrors Montoya Castillo’s findings linking WC and WtHR to cardiometabolic risk [23,25,26]. Feng et al. found BMI linked more to hypertension and WC to dysglycemia/dyslipidemia in northern China, with optimal cut-offs BMI 24 and WC 86/78 cm (men/women), showing both measures capture distinct but meaningful biological signals [27].

### Dyslipidemia – visceral fat signal and comparison of effect sizes

Dyslipidemia was common (536/678). WC showed the strongest correlation with triglycerides (rho 0.35), and WC/WtHR/BRI better predicted lipid abnormalities, while BMI predicted LDL and HDL less consistently. In ROC analyses, BMI and WC had acceptable discrimination (AUC BMI 0.749; WC 0.750), improving modestly with covariates (AUC 0.806). These patterns mirror meta-analyses showing WtHR outperforms BMI for diabetes, metabolic syndrome, and cardiovascular outcomes, and a Canadian cohort where WC, not BMI, strongly predicted serum lipids [28,29]. These quantitative patterns are consistent with meta-analytic evidence showing WtHR and WC frequently outperform BMI for lipid endpoints [5,22] and with longitudinal analyses where BMI and WC trajectories predict incident dyslipidemia, Zhang et al. found BMI/WC trajectories associated with later dyslipidemia with gut microbiota as a mediator [30]. Feng et al. similarly reported stronger WC-dyslipidemia associations than BMI [28]. WC’s strong association with triglycerides supports visceral fat’s role in delivering hepatic free fatty acids, driving an atherogenic, hypertriglyceridemic profile.

### FLD/NAFLD – where central indices and BRI shine

Hepatic outcomes showed the strongest anthropometric gradients: BMI increased from 24.0 to 30.0 kg/m² and WC from 86 to 104 cm (p < 0.001). Both BMI and WC strongly predicted FLD/NAFLD (AUC 0.753–0.777). BRI and WtHR are superior visceral fat indicators, showing higher NAFLD odds than ABSI. A meta-analysis (~60,000 participants) reported pooled BRI AUC 0.803, OR 2.87, supporting BRI’s value for liver-disease risk assessment beyond BMI or WC [31,32]. Still, WC alone captured much of the clinical signal, which argues for its pragmatic use as a first-line screening tool in clinical practice.

### Diabetes – limited discrimination by single anthropometrics

Anthropometry poorly discriminated diabetes (AUC BMI 0.543; WC 0.579), but adding demographics and diet improved AUC to ~0.736, highlighting the influence of age, sex, and lifestyle. Attenuation after adjustment has been noted by Seo et al. and others [26,33]. Prior cohort studies show that anthropometric indices’ predictive value for diabetes, hypertension, and lipid abnormalities drops after adjusting for age, sex, or other confounders – a pattern reflected in our data, where BMI and WC alone poorly discriminated diabetes (AUC 0.543–0.579) and associations with hypertension weakened after adjustment [34]. Mediation analyses show that 25–37% of the link between central adiposity (WC or WtHR) and diabetes is mediated by lipid abnormalities, highlighting that anthropometry alone may miss metabolic risk reflected in lipid measures [35]. Thus, anthropometry alone is insufficient for diabetes screening in many clinical populations – biochemical screening remains essential.

### Thyroid disease – a BMI-specific association

Our finding that BMI but not WC was higher in thyroid disease aligns biologically, as hypothyroidism raises weight via lower metabolic rate and fluid retention rather than visceral fat. A meta-analysis of 38 studies (49,427 individuals) reported large BMI increases (ES 1.092; 95% CI 0.755–1.429) but only moderate WC effects (ES 0.759; 95% CI 0.419–1.099) [36]. In a Chinese study of 193 with subclinical hypothyroidism, some thyroid-homeostasis indices were linked to BMI, but associations with WC and WtHR disappeared after adjusting for age, sex, metabolic status, and other confounders [37]. These sources support that thyroid dysfunction, particularly hypothyroidism, is more strongly linked to overall adiposity (BMI) than central obesity (WC), consistent with our finding that BMI but not WC was higher in our thyroid disease subgroup.

A study of Mexican Americans using SAFHS and NHANES 2007–2010 data found WC independently associated with a composite Thyroid Function Index, even after adjusting for BMI, age, sex, and other covariates; each SD increase in WC raised the odds of hypothyroidism [38]. Collectively, these findings reinforce that thyroid-adiposity associations differ across populations and disease phenotypes, but they provide empirical support for our observation that BMI retains unique clinical information for thyroid disease that is not captured by WC, and that BMI should not be abandoned solely in favor of central adiposity indices when evaluating endocrine risk.

### Prospective evidence and hard outcomes – WC matters for mortality when diabetes coexists

High WC (>94 cm) with diabetes strongly raised cardiovascular mortality risk (HR 3.78, 95% CI 3.35–3.98) in 1,521 adults over 9.2 years, adding prognostic value beyond traditional risk factors [28]. These data highlight that WC identifies a high-risk central-adiposity/metabolic phenotype. Similarly, Montoya Castillo showed WtHR predicts combined cardiometabolic burden (ORs ~ 2–3), capturing risk beyond single outcomes [23].

### Cut-offs – international convergence but population specificity

Across our and others’ studies optimal cut-offs cluster in the mid-20s for BMI and high-80s to low-90s cm for WC. In our cohort, Youden-derived thresholds concentrated near BMI ~ 25–26 kg/m² and WC ~ 88–93.5 cm (e.g., FLD: BMI 25.08, WC 88.5 cm; NAFLD: BMI 26.03, WC 93.5 cm). Suwała et al. proposed BMI 27.6 and WC 93/99 cm for SCORE2-based ICVR in a European cohort [24]; Montoya Castillo’s Colombian study found WtHR > 0.5 more strongly associated with risk than BMI. Adiposity measures included WtHR > 0.5, WC ≥ 80 cm (women)/ ≥ 90 cm (men), and BMI with overweight ≥25 kg/m² and obesity ≥30 kg/m² [23]. Asian studies (e.g., Taiwanese cohorts) show lower BMI/WC cut-offs due to ethnic differences in body composition and cardiometabolic risk. Optimal BMI ranged 24.5–25.7 kg/m² (men) and 22.6–24.0 kg/m² (women); WC 83.7–89.4 cm (men) and 73.5–80.4 cm (women), confirming Asians develop risk at lower thresholds than Caucasians [39]. These patterns support two practical points: (1) central indices are robust across settings, and (2) optimal numeric thresholds need local calibration for ethnic and age structure before wholesale adoption.

### Strengths and limitations

Strengths of our work include direct head-to-head comparison of BMI and WC (plus several derived indices) across multiple clinically relevant endpoints in a real-world hospital setting, and reporting of discrimination statistics (AUCs), odds ratios, correlation coefficients, and empiric cut-offs for each outcome. However, we were limited by the cross-sectional design (no incident events), possible under-ascertainment/misclassification for diabetes and early NAFLD, single-site hospital sampling limiting population generalizability, and residual confounding from lifestyle/socioeconomic variables imperfectly measured. Because hypertension, diabetes mellitus, and thyroid disease were classified using existing medical records and medication history without active diagnostic confirmation, some degree of underdiagnosis or misclassification, particularly for undiagnosed or early disease cannot be excluded.

## Conclusion

In this Nepalese population, BMI and waist circumference demonstrated complementary but distinct relationships with cardiometabolic risk, supporting the concept that conventional obesity markers may behave differently in our population. While BMI showed stronger odds for fatty liver disease, NAFLD, and dyslipidemia, waist circumference and central adiposity indices, particularly WtHR and BRI demonstrated superior specificity for triglyceride-related and hepatic metabolic abnormalities, suggesting that visceral adiposity may be a more clinically relevant determinant of cardiometabolic risk in Nepalese adults. Importantly, standalone anthropometric measures showed only fair discriminatory ability, whereas predictive performance improved substantially after incorporation of age and sex, emphasizing the need for multifactorial risk assessment rather than reliance on single obesity indices alone. These findings provide population-specific evidence supporting the routine use of waist circumference alongside BMI, while also considering patients’ age and sex during cardiometabolic risk assessment and screening.

## Supporting information

S1 Table Optimal cut-offs, sensitivities, and specificities of BMI and waist circumference for cardiometabolic risk factors. Table showing the results of cut-offs of BMI and waist circumference and their associated sensitivities and specificities of different cardiometabolic risk factors in our population.
(DOCX)

S1 File Raw data. Anonymized raw data used for the analysis and results of our study.
(XLSX)

## Abbreviations

ABSI: A Body Shape Index
AACE: American Association of Clinical Endocrinologists
ADA: American Diabetes Association
ALT: Alanine Aminotransferase
AMI: Acute Myocardial Infarction
AUC: Area Under the Curve
BMI: Body Mass Index
BRI: Body Roundness Index
CKD: Chronic Kidney Disease
CVD: Cardiovascular Disease
DM: Diabetes Mellitus
ECLIA: Electrochemiluminescence Immunoassay
EQAS: External Quality Assessment Services
FLD: Fatty Liver Disease
GP OPD: General Practice Outpatient Department
HDL: High-Density Lipoprotein
HTN: Hypertension
IFCC: International Federation of Clinical Chemistry
IOM: Institute of Medicine
IRC: Institutional Review Committee
LDL: Low-Density Lipoprotein
MCVTC: Manmohan Cardiothoracic Vascular and Transplant Centre
NAFLD: Non-Alcoholic Fatty Liver Disease
NCDs: Non-Communicable Diseases
OR: Odds Ratio
ROC: Receiver Operating Characteristic,
T3: Triiodothyronine
T4: Tetraiodothyronine
TC: Total Cholesterol
TG: Triglycerides
TSH: Thyroid-Stimulating Hormone
TUTH: Tribhuvan University Teaching Hospital
UA: Uric Acid
UV: Ultraviolet
WC: Waist Circumference
WBMIR: Waist-to-BMI Ratio
WHpR: Waist-to-Hip Ratio
WtHR: Waist-to-Height Ratio

## References

[1] Haldar S, Chia SC, Henry CJ. Body Composition in Asians and Caucasians: Comparative analyses and influences on cardiometabolic outcomes. Adv Food Nutr Res. 2015;75:97–154. doi: 10.1016/bs.afnr.2015.07.001 26319906

[2] Powell-Wiley TM, Poirier P, Burke LE, Després J-P, Gordon-Larsen P, Lavie CJ, et al. Obesity and cardiovascular Disease: A scientific statement from the American Heart Association. Circulation. 2021;143(21):e984–1010. doi: 10.1161/CIR.0000000000000973 33882682 PMC8493650

[3] Kidy FF, Dhalwani N, Harrington DM, Gray LJ, Bodicoat DH, Webb D, et al. Associations between anthropometric measurements and cardiometabolic risk factors in white European and South Asian adults in the United Kingdom. Mayo Clin Proc. 2017;92(6):925–33. doi: 10.1016/j.mayocp.2017.02.009 28578782

[4] Prasad DS, Kabir Z, Suganthy JP, Dash AK, Das BC. Appropriate anthropometric indices to identify cardiometabolic risk in South Asians. WHO South East Asia J Public Health. 2013;2(3):142–8. doi: 10.4103/2224-3151.206760 28615589

[5] Ashwell M, Gunn P, Gibson S. Waist-to-height ratio is a better screening tool than waist circumference and BMI for adult cardiometabolic risk factors: systematic review and meta-analysis. Obes Rev. 2012;13(3):275–86. doi: 10.1111/j.1467-789X.2011.00952.x 22106927

[6] Jayawardana R, Ranasinghe P, Sheriff MHR, Matthews DR, Katulanda P. Waist to height ratio: A better anthropometric marker of diabetes and cardio-metabolic risks in South Asian adults. Diabetes Res Clin Pract. 2013;99(3):292–9. doi: 10.1016/j.diabres.2012.12.013 23298662

[7] Williams R, Periasamy M. Genetic and Environmental Factors Contributing to visceral adiposity in Asian populations. Endocrinol Metab (Seoul). 2020;35(4):681–95. doi: 10.3803/EnM.2020.772 33397033 PMC7803598

[8] Ross R, Neeland IJ, Yamashita S, Shai I, Seidell J, Magni P. Waist circumference as a vital sign in clinical practice: A consensus statement from the IAS and ICCR working group on visceral obesity. Nat Rev Endocrinol. 2020;16(3):177–89. 32020062 10.1038/s41574-019-0310-7 PMC7027970

[9] Huang Y, Guo P, Karmacharya BM, Seeruttun SR, Xu DR, Hao Y. Prevalence of hypertension and prehypertension in Nepal: A systematic review and meta-analysis. Glob Health Res Policy. 2019;4:11. doi: 10.1186/s41256-019-0102-6 31165100 PMC6489280

[10] Bhattarai SA, Pyakurel M, et al. Cardiovascular disease trends in Nepal – An analysis of global burden of disease data 2017. IJC Heart & Vasculature. 2020;30:100602. 32775605 10.1016/j.ijcha.2020.100602 PMC7399110

[11] Wei J, Nie P, Gao L, Mi Y, Wang Y. Time trends and disparities of obesity and related national policies and programs in Nepal: a systematic review. Global Health Journal. 2024;8(2):46–57. doi: 10.1016/j.glohj.2024.05.006

[12] Sharma SK, Gautam U, Bhattarai U, Katuwal P, Perone SA, Zimmermann K. Cardiometabolic multimorbidity and associated risk factors in a cohort identified through community-based screening in Eastern Nepal. European Heart Journal. 2024;45(Supplement_1).

[13] Health Mo, Population/Nepal, New ERA, ICF. Nepal demographic and health survey 2022. Kathmandu, Nepal: Ministry of Health and Population [Nepal]. 2023.

[14] Khanal AG, Dahal I, Mishra S, Wasti SP, Ghimire R. Hypertension care cascade in Nepal: Findings from Nepal Demographic and Health Survey 2022. medRxiv. 2025(25323662).

[15] Cengiz M, Sentürk S, Cetin B, Bayrak AH, Bilek SU. Sonographic assessment of fatty liver: Intraobserver and interobserver variability. Int J Clin Exp Med. 2014;7(12):5453–60. 25664055 PMC4307502

[16] Expert Panel on Detection, Evaluation, and Treatment of High Blood Cholesterol in Adults. Executive Summary of The Third Report of The National Cholesterol Education Program (NCEP) expert panel on detection, evaluation, and treatment of high blood cholesterol in adults (adult treatment panel III). JAMA. 2001;285(19):2486–97. doi: 10.1001/jama.285.19.2486 11368702

[17] American Diabetes Association Professional Practice Committee. 2. Diagnosis and classification of diabetes: standards of care in diabetes-2024. Diabetes Care. 2024;47(Suppl 1):S20–42. doi: 10.2337/dc24-S002 38078589 PMC10725812

[18] Alper BS, Price A, van Zuuren EJ, Fedorowicz Z, Shaughnessy AF, Oettgen P, et al. Consistency of recommendations for evaluation and management of hypertension. JAMA Netw Open. 2019;2(11):e1915975. doi: 10.1001/jamanetworkopen.2019.15975 31755945 PMC6902818

[19] Shaver N, Beck A, Bennett A, Wilson BJ, Garritty C, Subnath M, et al. Screening for hypertension in adults: Protocol for evidence reviews to inform a Canadian Task Force on Preventive Health Care guideline update. Syst Rev. 2024;13(1):17. doi: 10.1186/s13643-023-02392-1 38183086 PMC10768239

[20] Hoermann R, Midgley JEM, Larisch R, Dietrich JW. Homeostatic control of the thyroid-pituitary Axis: Perspectives for diagnosis and treatment. Front Endocrinol (Lausanne). 2015;6:177. doi: 10.3389/fendo.2015.00177 26635726 PMC4653296

[21] Razvi S, Bhana S, Mrabeti S. Challenges in interpreting thyroid stimulating hormone results in the diagnosis of thyroid dysfunction. J Thyroid Res. 2019;2019:4106816. doi: 10.1155/2019/4106816 31662841 PMC6778876

[22] Browning LM, Hsieh SD, Ashwell M. A systematic review of waist-to-height ratio as a screening tool for the prediction of cardiovascular disease and diabetes: 0·5 could be a suitable global boundary value. Nutr Res Rev. 2010;23(2):247–69. doi: 10.1017/S0954422410000144 20819243

[23] Montoya Castillo M, Martínez Quiroz W de J, Suarez-Ortegón MF, Higuita-Gutiérrez LF. Waist-to-height ratio, waist circumference, and body mass index in relation to full cardiometabolic risk in an adult population from Medellin, Colombia. J Clin Med. 2025;14(7):2411. doi: 10.3390/jcm14072411 40217861 PMC11989366

[24] Suwała S, Junik R. Body mass index and waist circumference as predictors of above-average increased cardiovascular risk assessed by the SCORE2 and SCORE2-OP calculators and the proposition of new optimal cut-off values: Cross-sectional single-center study. J Clin Med. 2024;13(7):1931. doi: 10.3390/jcm13071931 38610696 PMC11012561

[25] Tran NTT, Blizzard CL, Luong KN, Truong NLV, Tran BQ, Otahal P, et al. The importance of waist circumference and body mass index in cross-sectional relationships with risk of cardiovascular disease in Vietnam. PLoS One. 2018;13(5):e0198202. doi: 10.1371/journal.pone.0198202 29813112 PMC5973604

[26] Seo D-C, Choe S, Torabi MR. Is waist circumference ≥102/88cm better than body mass index ≥30 to predict hypertension and diabetes development regardless of gender, age group, and race/ethnicity? Meta-analysis. Prev Med. 2017;97:100–8. doi: 10.1016/j.ypmed.2017.01.012 28137662

[27] Feng R-N, Zhao C, Wang C, Niu Y-C, Li K, Guo F-C, et al. BMI is strongly associated with hypertension, and waist circumference is strongly associated with type 2 diabetes and dyslipidemia, in northern Chinese adults. J Epidemiol. 2012;22(4):317–23. doi: 10.2188/jea.je20110120 22672914 PMC3798650

[28] Savva SC, Lamnisos D, Kafatos AG. Predicting cardiometabolic risk: waist-to-height ratio or BMI. A meta-analysis. Diabetes Metab Syndr Obes. 2013;6:403–19. doi: 10.2147/DMSO.S34220 24179379 PMC3810792

[29] Brenner DR, Tepylo K, Eny KM, Cahill LE, El-Sohemy A. Comparison of body mass index and waist circumference as predictors of cardiometabolic health in a population of young Canadian adults. Diabetol Metab Syndr. 2010;2(1):28. doi: 10.1186/1758-5996-2-28 20459858 PMC2883969

[30] Zhang X, Guan F, Gou W, Wang Q, Du S, Su C, et al. Multi-trajectories of BMI, waist circumference, gut microbiota, and incident dyslipidemia: A 27-year prospective study. mSystems. 2025;10(5):e0024325. doi: 10.1128/msystems.00243-25 40293249 PMC12090771

[31] Motamed N, Rabiee B, Hemasi GR, Ajdarkosh H, Khonsari MR, Maadi M, et al. Body roundness index and waist-to-height ratio are strongly associated with non-alcoholic fatty liver disease: A population-based study. Hepat Mon. 2016;16(9):e39575. doi: 10.5812/hepatmon.39575 27822266 PMC5091031

[32] Khanmohammadi S, Fallahtafti P, Habibzadeh A, Ezzatollahi Tanha A, Alamdari AA, Fallahtafti P, et al. Effectiveness of body roundness index for the prediction of nonalcoholic fatty liver disease: a systematic review and meta-analysis. Lipids Health Dis. 2025;24(1):117. doi: 10.1186/s12944-025-02544-3 40148946 PMC11948846

[33] Sardinha LB, Santos DA, Silva AM, Grontved A, Andersen LB, Ekelund U. A comparison between BMI, waist circumference, and waist-to-height ratio for identifying cardio-metabolic risk in children and adolescents. PLoS One. 2016;11(2):e0149351. doi: 10.1371/journal.pone.0149351 PMC4762486 26901828

[34] Abbasi F, Blasey C, Reaven GM. Cardiometabolic risk factors and obesity: Does it matter whether BMI or waist circumference is the index of obesity?. Am J Clin Nutr. 2013;98(3):637–40. doi: 10.3945/ajcn.112.047506 23885045 PMC3743728

[35] Lu S, Kuang M, Qiu J, Li W, Zhang M, Sheng G, et al. Lipids as the link between central obesity and diabetes: Perspectives from mediation analysis. BMC Endocr Disord. 2024;24(1):229. doi: 10.1186/s12902-024-01764-5 39468602 PMC11514969

[36] Kretli-Souza D, Paulino NE, Asevedo RA, Lemos SCL, Meireles-Oliveira G, Alves-Silva Y, et al. Body composition changes across a spectrum of hypothyroidism severity - A systematic review and meta-analysis. Rev Endocr Metab Disord. 2025;26(6):1037–50. doi: 10.1007/s11154-025-09988-z 40748421

[37] Zhou Y, Ke S, Wu K, Huang J, Gao X, Li B, et al. Correlation between thyroid homeostasis and obesity in subclinical hypothyroidism: Community-based cross-sectional research. Int J Endocrinol. 2021;2021:6663553. doi: 10.1155/2021/6663553 34135958 PMC8179776

[38] Mamtani M, Kulkarni H, Dyer TD, Almasy L, Mahaney MC, Duggirala R, et al. Increased waist circumference is independently associated with hypothyroidism in Mexican Americans: Replicative evidence from two large, population-based studies. BMC Endocr Disord. 2014;14:46. doi: 10.1186/1472-6823-14-46 24913450 PMC4057819

[39] Li W-C, Chen I-C, Chang Y-C, Loke S-S, Wang S-H, Hsiao K-Y. Waist-to-height ratio, waist circumference, and body mass index as indices of cardiometabolic risk among 36,642 Taiwanese adults. Eur J Nutr. 2013;52(1):57–65. doi: 10.1007/s00394-011-0286-0 22160169 PMC3549404

