# Supplementary material for: Waist circumference versus BMI: A cross-sectional study comparing Cardiometabolic Risk association in the Nepalese Population
Source: PLoS One. 2026 Jul 16;21(7):e0353676. doi: 10.1371/journal.pone.0353676 (PMC13375131; doi:10.1371/journal.pone.0353676)
Supplement: S1 Table — Table showing the results of cut-offs of BMI and waist circumference and their associated sensitivities and specificities of different cardiometabolic risk factors in our population. (DOCX) [file pone.0353676.s001.docx]

**Waist circumference versus BMI: Comparing Cardiometabolic Risk association in the Nepalese Population**

Sushan Gautam^1#^, Abhinav Bhattarai^2#^, Simran KC^1^, Roopal Singh^1^, Sanjit Sah^3^, Bishal Budha^1^,

Chandra Mani Poudel^4^, Raja Ram Khanal^4^

**Correspondence**

Dr. Sushan Gautam, MBBS

Department of Internal Medicine, Institute of Medicine, Tribhuvan University

Kathmandu, Nepal

Phone: +9779861288941

Email: [gsooshan@gmail.com](mailto:gsooshan@gmail.com)

***Table 1: Optimal cut-offs, sensitivities and specificities of BMI and WC for cardiometabolic risk factors***

| **Outcomes** | **Parameter** | **Cut-off** | **Sensitivity** | **Specificity** | **Youden’s J** |
| --- | --- | --- | --- | --- | --- |
| HTN | BMI | 24.09 | 88.7% | 39.2% | 0.278 |
|  | Waist circumference | 88.5 | 76.6% | 48.3% | 0.249 |
| Diabetes | BMI | 24.43 | 90.5% | 29.4% | 0.199 |
|  | Waist circumference | 87.5 | 78.6% | 41.4% | 0.200 |
| Thyroid disease | BMI | 25.95 | 64.4% | 55.4% | 0.198 |
|  | Waist circumference | 95.5 | 81.6% | 25.2% | 0.068 |
| Fatty liver disease | BMI | 25.08 | 78.7% | 61.9% | 0.406 |
|  | Waist circumference | 88.5 | 82.4% | 60.2% | 0.494 |
| NAFLD | BMI | 26.03 | 70.9% | 67.6% | 0.386 |
|  | Waist circumference | 93.5 | 64.3% | 76.5% | 0.408 |
| Dyslipidemia | BMI | 24.82 | 65.3% | 73.2% | 0.385 |
|  | Waist circumference | 85.5 | 74.8% | 57.8% | 0.326 |

*Units: BMI kg/m^2^, Waist circumference cm.*
